# Patients’ Experience of therapeutic footwear whilst living at risk of neuropathic diabetic foot ulceration: an interpretative phenomenological analysis (IPA)

**DOI:** 10.1186/1757-1146-7-16

**Published:** 2014-02-22

**Authors:** Joanne S Paton, Anne Roberts, Graham K Bruce, Jonathan Marsden

**Affiliations:** 1 FF21 Peninsula Allied Health Centre, School of Health Professions, Plymouth University, Derriford Road, Plymouth PL6 8BH, UK; 2 SF18 Peninsula Allied Health Centre, School of Health Professions, Plymouth University, Derriford Road, Plymouth PL6 8BH, UK; 3 Diabetic Centre Level 6 Derriford Hospital, Derriford Road, Plymouth PL6 8DH, UK; 4 FF22 Peninsula Allied Health Centre, School of Health Professions, Plymouth University, Derriford Road, Plymouth PL6 8BH, UK

**Keywords:** Adherence/compliance, Diabetes, Neuropathy, Footwear, Interpretative phenomenological analysis, Semi-structured interview, Qualitative

## Abstract

**Background:**

Previous work has found that people with diabetes do not wear their therapeutic footwear as directed, but the thinking behind this behaviour is unclear. Adherence to therapeutic footwear advice must improve in order to reduce foot ulceration and amputation risk in people with diabetes and neuropathy. Therefore this study aimed to explore the psychological influences and personal experiences behind the daily footwear selection of individuals with diabetes and neuropathy.

**Methods:**

An interpretative phenomenological analysis (IPA) approach was used to explore the understanding and experience of therapeutic footwear use in people living at risk of diabetic neuropathic foot ulceration. This study benefited from the purposive selection of a small sample of four people and used in-depth semi structured interviews because it facilitated the deep and detailed examination of personal thoughts and feelings behind footwear selection.

**Findings:**

Four overlapping themes that interact to regulate footwear choice emerged from the analyses: a) Self-perception dilemma; resolving the balance of risk experienced by people with diabetes and neuropathy day to day, between choosing to wear footwear to look and feel normal and choosing footwear to protect their feet from foot ulceration; b) Reflective adaption; The modification and individualisation of a set of values about footwear usage created in the minds of people with diabetes and neuropathy; c) Adherence response; The realignment of footwear choice with personal values, to reinforce the decision not to change behaviour or bring about increased footwear adherence, with or without appearance management; d) Reality appraisal; A here and now appraisal of the personal benefit of footwear choice on emotional and physical wellbeing, with additional consideration to the preservation of therapeutic footwear.

**Conclusion:**

For some people living at risk of diabetic neuropathic foot ulceration, the decision whether or not to wear therapeutic footwear is driven by the individual ‘here and now’, risks and benefits, of footwear choice on emotional and physical well-being for a given social context.

## Background

Diabetes is a growing epidemic [1]. In 2000, diabetic disease was estimated to affect 171 million people worldwide, this figure is expected to increase to 366 million by 2030 [2]. Foot ulceration is a common and debilitating complication of diabetes affecting 15% of diabetic individuals at some time [3]. Living with foot ulceration has been linked to diminished wellbeing, quality of life and physical health [4,5]. The long-term outcome of foot ulceration is devastating, a substantial proportion of diabetic persons (11-26%) developing foot ulceration progress to amputation [6,7].

Diabetic peripheral neuropathy has been associated with increased ulceration risk [8]. It is recommended that people with diabetes and neuropathy wear insoles accommodated within therapeutic footwear to reduce mechanical tissue stress on the plantar surface of the foot and help in the prevention of foot ulceration [9]. However footwear and insoles are only effective in reducing ulcer risk when worn. Chantelau and Haage [10] report that neuropathic patients, with diabetes and a history of foot ulceration, wearing protective shoes for more than 60% of the daytime reduced ulcer relapse rate by 50%. However, a number of empirical studies found that although participants were wearing the prescribed footwear regularly, they were doing so for only part of the day [11-13]. Insole and footwear compliance must be improved to enable the person with diabetic neuropathy to accumulate sufficient wear time to potentially achieve therapeutic effect. The reasoning behind this selective therapeutic footwear use is unknown. There has been little focus on understanding how the lived experience of people with diabetes and neuropathy who have been provided therapeutic footwear influences footwear selection. If we can better understand the lived experience [14] of people with diabetes and neuropathy who are issued with therapeutic footwear and insoles and how they use their footwear, then we can design more effective implementation strategies to support adherence to therapeutic footwear advice.

Quantitative research investigating adherence to footwear advice in diabetic people suggests that a large number of people are not wearing their therapeutic footwear all day, every day as directed [13]. Several surveys have shown that people chose not to wear their therapeutic footwear because; the therapeutic footwear did not appear stylish enough (to the women subjects); they had a preference for their own shoes, the footwear was reserved for use on special occasions; or they preferred not to wear therapeutic footwear whilst indoors, despite footwear education to the contrary [11,12,15]. The differing perspectives of patients and health professionals on the use of therapeutic footwear have been explored to begin to explain this apparent knowledge-action gap [15]. Health professionals focused on morbidity limitation with no regard for the difficulties experienced by patients in adhering to advice. Whereas for diabetic people within the lived experience, the advice received required behaviour change which they considered difficult and beyond what was socially and personally acceptable. The way in which people think about and react to the professional recommendation to wear their therapeutic footwear all of the time is likely to be a uniquely personal experience. Understanding this personal perspective in detail is essential for informing footwear education in the clinical setting [16].

Most literature focuses on the gap between patient knowledge and patient behaviour in those with diabetes who are required to self-manage their blood sugar control [16-18]. As the burden of diabetic complications, including neuropathic foot ulceration, begins to outweigh that of glycaemic control some focus has shifted toward diabetic foot ulceration prevention [19]. Despite foot ulceration being preventable and there being a shift toward more patient centred approaches to ulcer prevention, a recent Cochrane review of randomised control trials found little evidence of a reduction in foot ulceration rate following a programme of patient education [20]. Vileikyte et al 2004 suggest that attention should now be directed toward the psychological influences and personal experiences affecting footwear daily life routines [5]. Work in this area is limited, although some progress has been made to explore how the beliefs, emotions and experiences of people living under the threat of diabetic foot ulceration influences their behavioural responses [5,21,22]. The authors [5,21,22] concluded that patient beliefs about how foot ulcers are caused, and their emotional response toward foot ulceration including fear and worry, influence their foot care behaviour [5,21]. Although relevant, it is difficult to understand specifically how these findings relate to the individual thinking behind the daily footwear selection of patients choosing not to wear their insoles and footwear for all of the daytime.

The aim of this study was to explore the understanding and experiences of people living with diabetes and neuropathy in relation to wearing insoles and therapeutic shoes, and to explore how this might explain insole and footwear wearing habits. In particular we used a qualitative method grounded in interpretative phenomenological analysis (IPA), because this method is suitable for understanding the unique personal perspectives and meanings associated with living at risk of diabetic neuropathic foot ulceration, and use or non-use of therapeutic footwear [23].

## Method

We required an inductive approach and chose IPA to both capture and analyse the data. IPA is committed to understanding how people make sense of their lived experience, particularly when the everyday becomes significant. It is therefore well suited to exploring what happens when people at risk of foot ulceration make decisions about how to use footwear specifically designed to protect their feet from damage. IPA allows consideration of the lived experience of the person being researched and also the researcher’s experiences through a process of reflexivity [23]. The participant interprets their own situation and the researcher in turn uses their knowledge and experience to interpret the meaning of the data given to them by the participant (double hermeneutic).

### Participants

People attending NHS podiatry clinics within South West England were purposively sampled. People were invited to join the study if they had diabetes and neuropathy (as defined by clinical testing using a monofilament and tuning fork) and if they had been supplied with therapeutic insoles and footwear at least 6 months ago. Those interested in joining the study were provided an information sheet about the study along with the contact details of the researchers. People volunteering to join the study then contacted the researcher for additional verbal information, to ask questions and to be pre-screened against the inclusion criteria. All four participants meeting the inclusion criteria gave written consent to participate in interviews.

The two men aged 58 and 71 years and two women, aged 59 and 84 years all described their current foot health as fine or good. Two of the participants worked full time, one was retired and the other although retired still worked on a part-time basis. The women participants lived with their spouses, the two men lived alone. Two had been living with diabetes for more than 20 years and two between 8-10 years. All had experience of wearing therapeutic insoles and shoes for between 4 and 10 years. All recalled some history of foot problems, ranging in severity from blistering to bilateral Charcot Arthropathy and the amputation of a digit. Participant’s names have been changed to protect the identity of the person.

### Procedure

We obtained ethical approval from the South West Regional Ethical Committee before study commencement. One researcher (JP) conducted and audio-recorded in-depth semi-structured interviews within the participant’s own home or a place preferable to them, such as the local hospital or university setting. Interviews lasted up to one hour; each began by asking participants about general demographic information such as age, duration of diabetes and any self-reported foot problems. In line with IPA, questions focused on each participant’s unique experience of how therapeutic footwear fitted with daily life and their thoughts and feelings about wearing therapeutic footwear. As such the interview schedule (Additional file 1) was treated as a guide to allow the flow of conversation to follow the particular concerns of the participant. The schedule included a number of probes designed to prompt the participant into disclosing an increased level of depth and detail.

The audio recording was transcribed verbatim and analysed, following the strategy recommended for IPA to theme and interpret the data [23]. One researcher (JP) undertook a line by line interpretative analysis of the concerns and understandings of each participant to produce a comprehensive analysis to make sense of the personal meaning within each account. During the next stage these initial notes were used to identify within case themes of commonality and differences. Drawing on her own thoughts and perceptions, and through an iterative process of reflection, the researcher (JP) developed a structure of superordinate themes for each case, to explore relationships between themes. This interpretative viewpoint was influenced by her professional role as a podiatrist and experience of fitting and assessing therapeutic footwear. Throughout the analysis a reflective account of the interpretative thought processes was recorded and those thoughts balanced with discussions held with the second author (AR not a podiatrist). Once this process had been repeated for each case the process moved to identify patterns across cases. Finally higher order concepts were configured to illustrate both unique idiosyncratic and shared characteristics of the study participants.

The second author (AR) audited the first two transcripts to check for credibility between the annotations, the list of themes and the original transcript. Concordance was achieved through discussion based on the original transcript. The second case was presented to a steering group of healthcare professionals with qualitative research experience and their personal perspectives discussed. One participant was invited to reflect and comment on the final interpretation of her thoughts and feelings to ensure she considered the findings relevant and accurate.

## Findings

The analysis identified four recurrent and related themes that together revealed a process which was rehearsed frequently within the minds of the participants when making the decision of when and where to wear their therapeutic footwear and insoles. It became clear from the analysis that the decision to wear the footwear was not simply a case of interpreting the footwear advice given, but was based on a complex interaction of internal thoughts and values, and external events that developed and fluctuated. The four themes derived from the participants’ decision making processes were; 1) the self-perception dilemma (do I appear normal versus am I at risk of foot ulceration?); 2) reflective adaption (the adaptation of personal values regulating footwear choice); 3) adherence response (the alignment of therapeutic footwear adherence with personal values); 4) reality appraisal (the here and now impact of footwear choice).

Below we explore the understandings and experiences of four people living with diabetes and neuropathy issued with insoles and therapeutic shoes, and how their understandings and experiences explain insole and footwear wearing habits. We concentrate in detail on the shared themes but also the participant’s individual accounts to conceptualise their individual footwear decision making.

### The self-perception dilemma: do I appear normal versus am I at risk of foot ulceration?

When first issued with therapeutic footwear, all participants assessed the visual appearance to determine if the style of the shoe fitted with their perception of the accepted ‘norm’. Derek, for example, views the appearance of his therapeutic footwear as meeting his individual vision of a men’s winter shoe: ‘I mean, they look like normal shoes don’t they’.

In sharp contrast the women participants believed that the style of their therapeutic footwear was far removed from their internalised image of a ladies shoe. Barbara initially remembers the repercussions of being given what she viewed as ‘horrible’ footwear:

“Um I just couldn’t believe that I was just given these shoes. I should of taken a picture because they were like old diving boots and I thought I [pause] ya know I wana work, I wana be relatively normal and do the things that I wana do I just thought I can’t do that with these horrible [pause] shoes”.

Balanced against this assessment of normality was the question of foot health risk. Participants generally were aware that the footwear was provided for therapeutic benefit. Participants had a varying understanding of the potential for diabetic foot ulceration, dependant largely on personal circumstance, reason for the initial referral and any subsequent foot care education. However the key determining factor for participants resolving the conflict between achieving social inclusion and minimising risk of foot ulceration was whether or not they felt they were at immediate risk of foot ulceration and the estimated magnitude of that risk.

Derek: “I don’t buy shoes at all. I’ve only got two pairs of shoes those and the ones that the orthotist gave me probably three weeks and month ago. I wouldn’t consider going into a shoe shop and buying shoes. Ya know black or whatever. Umm but I wouldn’t I would only wear shoes that the orthotist has got for me”.

[and why is that? why would you only wear those?]

“I’d be worried about rubbing toes and perhaps having another toe removed or worse ya know”.

### Reflective adaption: adaptation of personal values regulating footwear choice

The intention to wear therapeutic footwear all of the time was not sufficient to alter behaviour. For participants to make the decision to wear their therapeutic footwear and then find the motivation to change behaviour, they first needed a set of personal regulating values to measure their actions against. These personal values were individually constructed and continuously adapted through self-reflection of a number of influencing factors including;

#### Self-image

Thoughts about self-image were gender dependant, predominantly a concern for the women participants. Overtime both women have at some level adapted their self-image to take account of the therapeutic footwear. During that transitional process both women have adjusted their personal values to become more comfortable with their self-image. Edith demonstrated a change in mind set when faced with an important social gathering ‘ I don’t take so much notice now…..it doesn’t bother me so much …’.

#### Everyday function

The benefit of maintaining function, and being considered by others as functionally normal, often became more important than negative issues relating to self-image, particularly for the women participants. The overriding concern for participants was to function within the social norm, and to that end, be seen to lead a normal life. Edith sums up her functional reliance on her footwear; ‘without these [therapeutic] shoes I don’t think I’d ever go out’. Likewise it appeared that through a reflective process, any visual implications of the therapeutic footwear with regard to obvious disability were overridden by Barbara’s desire to lead a functionally normal life, ‘If I didn’t have shoes that I could wear and walk around in I couldn’t function’.

#### Environment and activity risk

Day to day the male participants in particular regularly appeared to use a process of reflection to reset their personal values and determine the risk they were about to put their feet under, given a particular social context or planned activity. This reflection often seemed almost intuitive and situated in the here and now. Within a high risk setting the men were more likely to choose to wear their therapeutic footwear. However in environments considered to pose low risk to their foot, a rationale for an alternative footwear selection would often be made; ‘the orthotist tells me not to wear the sandals….because of injuries perhaps? But I don’t feel there’s a risk (at home)’. For both men participants, the level of risk was considered proportionate to the familiarity of the surroundings.

#### Pivotal event

Therapeutic footwear is issued to people with diabetic peripheral neuropathy as part of the foot ulcer prevention strategy, often before the recipient has experienced any symptoms. However Derek and Barbara described the impact of a specific life changing event where they experienced first-hand the consequences of diabetic neuropathic foot pathology. Once the consequence of taking risks with their choice of footwear had been realised, participants responded by reflecting on the event to identify in their minds a controllable cause. Where footwear was seen as contributory, participants took direct action to change their footwear choice to prevent reoccurrence; Derek ‘I’m frightened of wearing other shoes because of the problems before’.

### Adherence response: alignment of therapeutic footwear adherence with personal values

If current behaviour was aligned to personal values, then the footwear selection would remain unchanged and unchallenged in the minds of the participants. For example, Andrew suggested that the decision to wear slippers in preference to therapeutic shoes whilst indoors seemed common sense and a matter of course:

“Well I don’t really need to, if I want to go to the toilet I don’t really need to put me shoes on to go up the stairs do I ya know, also if I’m a like gona go up and have a shower”.

In contrast, Barbara recounts a pivotal moment in her transition to accepting her footwear when she made a conscious decision to align her behaviour to her newly formed set of values regarding her footwear selection:

“I’m never going to be able to wear this (retail shoes), so I did I parcelled them up and I took them down to [the charity shops] and thought right that’s the end of it”.

### Reality appraisal: the here and now impact of footwear choice

Participants reflected upon their self-perception, before selecting the footwear deemed suitable for a given situation. Finally participants would undertake a self-appraisal to review the reality of their footwear selection decision on their immediate sense of wellbeing. Three areas of impact were mentioned as of particular concern to participants:

a) The effect of footwear choice on physical health, including ability to work, attend social functions and shop without limitations. The majority of participants experienced a direct improvement in mobility and an increased sense of freedom whilst wearing their therapeutic footwear:

b) The effect of footwear choice on sense of wellbeing. Participants lived in the moment, responding to and being influenced by their immediate feelings of wellbeing. In particular participants described an association between their footwear choice and mood. For most participants the act of releasing their feet from their therapeutic footwear was important, particularly whilst in the comfort of their own home, signalling a wind down transition towards ‘feeling more relaxed’. Edith summarises her views: ‘*But sometimes you feel you want to take the ordinary shoe off ya know to relax your foot’.*

c) The effect of footwear choice on the condition of their therapeutic footwear. Participants generally regarded their therapeutic footwear as an item of quality and value. This regard for their therapeutic footwear as a valued possession to be cherished fuelled an unhealthy desire to extend the useful life of the footwear and keep them in good condition. Edith recounted her rationale for her daily footwear choices:

Edith: “But it’s just one of these things I rather be comfortable because I mean I used to be in agonies trying to walk we couldn’t go for very long walks could we, because I just couldn’t….Because after I had the shoes and I bought this one (walking frame) and I said to (my husband) I’m sure I could go into town now cause for years we hadn’t gone to town had we”.

“…..winter or wet these, ‘cause I don’t want to spoil the white ones. I only put these [worn out condemned therapeutic footwear] on to save these [current therapeutic footwear] I won’t wear these [current therapeutic footwear] all day cause of wearing them out”.

## Discussion

By exploring the understandings and experiences of patients with diabetes and neuropathy issued with insoles and therapeutic shoes using an IPA approach, we shed light how an individual’s thoughts and feelings influence footwear choice, particularly the decision only to wear therapeutic footwear for some of the daytime.

### The self-perception dilemma: do I appear normal versus am I at risk of foot ulceration

Differences in psychological impact of therapeutic footwear appearance between men and women with diabetes and neuropathy might be explained by inference to work by Kaiser and colleagues [24]. This work exploring the clothing choice of disabled students used a negotiated outcomes perspective to combine the findings from focus groups with responses from open ended questionnaires. They concluded that disability was only disruptive when taken for granted social norms were breached, that is when responders looked different to everyone else [24]. Authors found that wearing special clothing was avoided because it reinforced differences between disabled and abled bodied persons, thus disabled students manipulated appearance by selecting normative clothing. In the current study women with diabetes and neuropathy found wearing the visibly different therapeutic footwear drew attention to an otherwise hidden disability, disrupting the taken for granted social norm.

Participants in this study described the daily life-disease conflict encountered between adhering to the social norm and responding to their estimate of ulceration risk when selecting footwear for a given circumstance. This dilemma between taking part in a normal life and managing the consequences of diabetes has been discussed in terms of other aspects of diabetic management [16,18]. Described in the current study as the self-perception dilemma, Beattie and colleagues [21] observed similar behaviour (termed ‘strategic adherence’), in a comparable patient group. Patients living at heightened risk of re-ulceration reported self-negotiating a comparable compromise between choosing to live a normal life or following foot care advice.

The self-perception dilemma played out in the minds of people with diabetes and neuropathy, particularly women, may be reduced if a resolution between fitting in with everyone else and protecting their feet from foot ulceration could be established. This gap might be reduced by normalising the appearance of the therapeutic footwear, for example where appropriate replicating high street fashion trends.

### Reflective adaption: adaptation of personal values regulating footwear choice

Good intention was not sufficient in itself to alter footwear behaviour. Seated within the Social Cognitive Theory of Self-Regulation framework [25], for participants to change behaviour and choose to wear their therapeutic footwear, they first needed to construct a set of personal regulating values against which to measure their actions. Participants reflected on a number of important personal experiences and emotional responses on which they assimilated a unique set of values about their therapeutic footwear and its use. Four important areas of influence framed within the here and now, formed the foundation on which these values were built; adjustment of self-image, ability to function day to day, perception of environment and activity risk, and experience of a pivotal event involving threat to foot health.

The findings of this study support the conceptual model of adherence to foot care developed by Vileikyte and colleagues [5] for patients with diabetic neuropathic foot complications. Patients construct common sense beliefs about their condition and its consequences that are fundamental in guiding their healthcare actions or more specifically footwear choice [5]. Based on the common sense model of adaption to chronic illness, Vileikyte and colleagues [5] identified that tangible experience and disease reality (the realisation of the personal psychosocial and physical consequences of ulcer development), rather than far removed theoretical ideals about preventative foot care management (health professional dominant footwear advice), elicit an emotional response strong enough to motivate a change in the personal beliefs regulating preventative foot care action.

### Adherence response: alignment of therapeutic footwear adherence with personal values

Like women with rheumatoid arthritis, women with diabetic neuropathy express concerns around the impact of their therapeutic footwear on self-image [26]. Both Edith and Barbara described a transition toward an acceptance of therapeutic footwear together with the adjustment of self-image. The women appeared to gradually shape a growing set of values supporting therapeutic footwear adherence. Each then described a gestalt moment when a conscious decision was made to commit to increased therapeutic footwear adherence.

The long transition toward therapeutic footwear acceptance, particularly for the women, might parallel the holistic on-going adjustment to living with diabetes and its implications [27]. Walker and colleagues [27] describe how learning to adjust to an illness like diabetes and its implications, is a process that constantly changes over time.

Both Edith and Barbara developed a coping strategy of therapeutic footwear concealment from self or others to dampen the psychological impact of choosing to wear therapeutic footwear. Barbara preferred to camouflage her footwear against the backdrop of her outfit, through careful colour blending. Alternatively Edith purposely obscured her footwear from herself whilst looking in the mirror. A similar concealment tactic has been observed in persons with coping with visual disability so they might present a normative appearance and maintain a positive sense of self [24,28]. To better support women in their transition to toward therapeutic footwear acceptance, practical advice on the management of appearance to conceal or deflect attention away from the therapeutic footwear might prove helpful.

### Reality appraisal: the here and now impact of footwear choice

The results of this study are aligned to the Social Cognitive Theory [25]. As part of the complex multi-faceted self-regulation mechanism, people assess factors that affect their sense of physical and emotional wellbeing through a systematic process of trial and error within their daily lives [25].

Despite talking of their initial visual dislike for therapeutic footwear, both Edith and Barbara acknowledged improved physical functioning whilst wearing them. The importance of functional normalcy eventually outweighed concerns regarding visual impact. Similarly when prostheses users were asked to prioritise between aesthetics and function, whereas new users felt normal appearance was most important, their views changed and over time functional normalcy took precedence [29].

Participants talked of a here and now connection between footwear choice and emotional state. The removal of footwear whilst indoors was seen as a home comfort, providing a psychological prompt to achieving a more relaxed and comfortable state. Participants talked in specific terms about a desire to release or relax their feet to help them unwind, inferring an inextricable real-time link between footwear and sense of wellbeing. Previous work suggested people with neuropathy and diabetes simply take off their shoes off whilst indoors despite footwear education to the contrary [30,31]. This study provides detail of the thoughts and feelings driving this apparent knowledge-action gap. The decision to remove therapeutic footwear whilst indoors is a complex interaction of perceived immediate risk, sense of wellbeing and a common sense method of preserving therapeutic footwear for outdoor use, positioned within everyday reality.

This study supports the need for a house shoe or slipper [11,30]. However to meet the needs of people with diabetes and neuropathy and maximise adherence, we recommend that the product should give the impression of home comfort, be quick and easy to put on, suitable for use after bathing, relatively disposable and readily available.

As with all qualitative research, the findings of this study cannot be generalised to the wider population. Rather the intentional use of a small but specific sample is suited to providing a greater depth of understanding and insight into individual idiosyncrasies. The aim of qualitative research is to improve our understanding of complex issues; it is not concerned with prevalence or incidence. Therefore when using IPA to fully explore in detail the particular thoughts and feelings of the people purposively sampled it was important that the sample size remained small scale in order to do justice to the rich and plentiful data. The findings are useful because they relate to and resonate with people from a similar population sharing a similar life experience. Understanding individual perceptions about footwear usage is of particular value in informing the generation of new ideas to improve footwear adherence strategies targeted at people with diabetes.

### Practical implications

Participants’ decisions to choose to wear therapeutic footwear were influenced by their perception of immediate risk of ulceration. Rather than talking to diabetic people about adhering to footwear advice it might be helpful for professionals and diabetic people to reflect on how they might use their therapeutic footwear to control and reduce the risk of foot ulceration. Participants were also more inclined to choose to wear their therapeutic footwear if they perceived it to benefit their personal wellbeing. Wearing therapeutic footwear might be more acceptable to people with diabetes and neuropathy if it is not viewed as an additional burden of their condition, but instead as a vehicle for functional normalcy.

Although this was a small sample, careful and detailed identification of the personal perspectives and meanings associated with living at risk of diabetic neuropathic foot ulceration, offers the potential to theorize about how therapeutic footwear might be improved for those particular people and those like them.

Therapeutic footwear design could be made more desirable if normalised to more closely reflect high street fashion trends (for example incorporating on trend colours and detailing), whilst maintaining the therapeutic objective. Current basic footwear education might be enhanced through the use of expert patients with personal experience of foot ulceration, who can talk through the implications of foot ulceration and share their thinking behind the decision to wear therapeutic footwear. Practical tips on appearance management might make therapeutic footwear appear more feasible to women. For example, the use of concealment or deflection techniques to reduce the visual impact of therapeutic footwear on their self-image, might be worthy of consideration. We recommend that the provision of therapeutic house shoes or slippers, in addition to therapeutic footwear, might better meet the needs of people with diabetes and neuropathy. However, to be useful, we believe that the product would need to meet the following criteria; lightweight, made of comforting materials, quick and easy to put on, suitable for use after bathing, relatively disposable and readily available.

### Future research

Given the importance of the use of recommended therapeutic footwear in reducing the risk of neuropathic foot ulceration, future research is needed to explore the transferability of these results. We suggest that the in-depth information gained from this small study could be useful for informing the design of a larger scale study. Moreover further work is needed to develop an individualised therapeutic footwear provision and education strategy. Research in this area would benefit from focusing on empowering patients to use therapeutic footwear as foot protection in the ‘here and now’.

## Conclusion

The personal values driving participants’ adherence to wearing therapeutic footwear constantly evolved as they adapted to the daily reality of living with diabetic peripheral neuropathy. Participants were aware of the need to protect their feet from damage by using therapeutic footwear, but they chose to moderate and personalise their adherence behaviour to fit their perceived level of ulceration risk, to save their footwear from wear and tear, to enhance their sense of physical and emotional wellbeing, and to maintain functional and social normalcy.

## Competing interest

The authors declared no potential conflicts of interest with respect to the research, authorship, and/or publication of this article.

The authors disclosed receipt of the following financial support for the research of this article: A CAT Clinical Lectureship Fellowship awarded by the National Institute of Health Research.

## Authors’ contribution

JP conceived and designed the study, conducted the interviews, extracted and analysed the data and produced the first and final draft. AR participated in the development of the study design, the interpretation and analysis of data, the review of academic content, the production of the initial draft and edited the final draft. GB and JM participated in the development of the research design, critically reviewed the academic content and participated in producing the final draft. All authors read and approved the final manuscript.

## Authors’ information

JP is a NIHR Clinical Research Fellow and Podiatrist at the University of Plymouth, UK, AR is an Associate Professor in Occupational Therapy at the University of Plymouth, UK, GB, is a Diabetes Specialist Podiatrist at Derriford Hospital Plymouth, UK, JM, is a Professor and Chair in Rehabilitation at the University of Plymouth, UK.

## Supplementary Material

Additional file 1 Interview schedule. Click here for file

## References

[B1] Chen L Magliano DJ Zimmet PZ The worldwide epidemiology of type 2 diabetes mellitus-present and future perspectives Nat Rev Endocrinol 2012 8 4 228 236 22064493 10.1038/nrendo.2011.183

[B2] Wild S Roglic G Green A Sicree R King H Global prevalence of diabetes. Estimates for the year 2000 and projections for 2030 Diabetes Care 2004 27 5 1047 1053 10.2337/diacare.27.5.1047 15111519

[B3] Singh N Armstrong DG Lipsky BA Preventing foot ulcers in patients with diabetes J Am Med Assoc 2005 293 2 217 228 10.1001/jama.293.2.217 15644549

[B4] Goodridge D Trepman E Embil JM Health-related qualtiy of life in diabetic patients with foot ulcers: literature review J Wound Ostomy Continence Nurs 2005 32 6 368 377 10.1097/00152192-200511000-00007 16301902

[B5] Vileikyte L Rubin R Leventhal H Psychological aspects of diabetic neuropathic foot complications: an overview Diabetes Metab Res Rev 2004 20 Suppl 1 S13 S18 15150807 10.1002/dmrr.437

[B6] Aldler A Boyko E Ahroni J Smith D Lower extremity amputation in diabetes; the independent effects of peripheral vascular disease, sensory neuropathy and foot ulcers Diabetes Care 1999 22 1029 1035 10.2337/diacare.22.7.1029 10388962

[B7] Ramsey S Newton K Blough D McCulloch D Sandhu N Reiber G Wagner E Incidence, outcomes and cost of foot ulcers in patients with diabetes Diabetes Care 1999 22 3 382 387 10.2337/diacare.22.3.382 10097914

[B8] Abbott C Vileikyte L Williamson S Carrington A Boulton A Multicenter study of the incidence of and predictive risk factors for diabetic neuropathic foot ulceration Diabetes Care 1998 21 7 1071 1075 10.2337/diacare.21.7.1071 9653597

[B9] Paton J Bruce G Jones R Stenhouse E Effectiveness of insoles used for the prevention of ulceration in the neuropathic diabetic foot: a systematic review J Diabetes Complicat 2011 25 1 52 62 10.1016/j.jdiacomp.2009.09.002 19854075

[B10] Chantelau E Haage P An audit of cushioned diabetic footwear: relation to patient compliance Diabet Med 1994 11 1 114 116 10.1111/j.1464-5491.1994.tb00240.x 8181241

[B11] Macfarlene D Jensen J Factors in diabetic footwear compliance J Am Podiatr Med Assoc 2003 93 6 485 491 14623991 10.7547/87507315-93-6-485

[B12] Knowles E Boulton A Do people with diabetes wear their prescribed footwear? Diabet Med 1996 13 1064 1068 10.1002/(SICI)1096-9136(199612)13:12<1064::AID-DIA253>3.0.CO;2-# 8973889

[B13] Waaijman R Keukenkamp R de Haart M Polomski W Nollet F Bus S Adherence to wearing prescription custom-made footwear in patients with diabetes at high risk for plantar foot ulceration Diabetes Care 2013 36 6 1613 1618 10.2337/dc12-1330 23321218 PMC3661819

[B14] Manen M van Researching the Lived Experience 1990 New York: SUNY Press

[B15] Johnson M Newton P Goyder E Patient and professional perspectives on prescribed therapeutic footwear for people with diabetes: a vignette study Patient Educ Couns 2006 64 1–3 167 172 16469472 10.1016/j.pec.2005.12.013

[B16] Zoffmann V Kirkevold M Realizing empowerment in difficult diabetes care: a guided self-determination intervention Qual Health Res 2012 22 1 103 118 10.1177/1049732311420735 21876206

[B17] Sulway M Tupling H Webb K Harris GF New techniques for changing compliance in diabetes Diabetes Care 1980 3 1 108 111 10.2337/diacare.3.1.108 7408599

[B18] Rosenbek Minet L Lonvig E Henriksen J Wagner L The experience of living with diabetes following a self-management program based on motivational interviewing Qual Health Res 2011 21 8 1115 1126 10.1177/1049732311405066 21471428

[B19] Gordis A Scuffham P Shearer A Oglesby A Tobian J The health care costs of diabetic peripheral neuropathy in the US Diabetes Care 2003 26 6 1790 1795 10.2337/diacare.26.6.1790 12766111

[B20] Dorresteijn J Kriegsman D Assendelft W Valk G Patient education for preventing diabetic foot ulceration Cochrane Database Syst Rev 2010 12 5 doi: 10.1002/14651858.CD001488

[B21] Beattie A Campbell R Vedhara K ‘What Ever I do its a lost cause.’ The emotional and behavioural experiences of individuals who are ulcer free living with the threat of developing further diabetic foot ulcers: a qualitative interview study Health Expect 2012 20 Advanced online publication. doi: 10.1111/j.1369-7625.2012.00768 10.1111/j.1369-7625.2012.00768.x PMC5060729 22429399

[B22] Gale L Vedhara K Searle A Kemple T Campbell R Patients perspectives on foot complications in type 2 diabetes: a qualitative study Br J Gen Pract 2008 58 553 555 563 10.3399/bjgp08X319657 18682014 PMC2566520

[B23] Smith J, Flowers P, Larkin M Interpretative Phenonmenological Analysis Theory Method and Research 2011 London: SAGE pulications Ltd

[B24] Kaiser S Freeman C Wingate S Stigmata and negotiated outcomes: management of appearance by persons with physical disabilities Deviant Behav 1985 6 2 205 224 10.1080/01639625.1985.9967670

[B25] Bandura A Social cognitive theory of self-regulation Organ Behav Hum Decis Process 1991 50 248 287 10.1016/0749-5978(91)90022-L

[B26] Williams A Nester C Ravey M Kottink A Klapsing M Women’s experiences of wearing therapeutic footwear in three European countries J Foot Ankle Res 2010 doi:10.1186/1757-1146-3-23 10.1186/1757-1146-3-23 PMC2959012 20932291

[B27] Walker J Jackson H Littlejohn G Models of adjustment to chronic illness: using the example of rheumatiod arthritis Clin Phychol Rev 2004 24 461 488 10.1016/j.cpr.2004.03.001 15245831

[B28] Kaiser S Wingate S Freeman C Chandler J Acceptance of physical disability and attitudes toward personal appearance Rehabil Psychol 1987 32 1 51 58

[B29] Murry C Being like everybody else: the personal meanings of a prosthesis user Disabil Rehabil 2009 31 7 573 581 10.1080/09638280802240290 19034778

[B30] Knowles EA Boulton AJ Do people with diabetes wear their prescribed footwear? Diabet Med 1996 13 12 1064 1068 10.1002/(SICI)1096-9136(199612)13:12<1064::AID-DIA253>3.0.CO;2-# 8973889

[B31] Knowles A The role of pressure relief in diabetic foot problems Diabet Foot 1998 1 2 55 63

