# Supplementary material for: Patients’ Experience of therapeutic footwear whilst living at risk of neuropathic diabetic foot ulceration: an interpretative phenomenological analysis (IPA)
Source: J Foot Ankle Res. 2014 Feb 22;7:16. doi: 10.1186/1757-1146-7-16 (PMC3937231; doi:10.1186/1757-1146-7-16)
Supplement: Additional file 1 — Interview schedule. [file 1757-1146-7-16-S1.docx]

**Additional file 1. V2 07/12/11**

**Additional file 1. Interview Schedule**

**The Interview Schedule**

Area to be talked: the experience of wearing orthopaedic insoles and footwear

Range of topic areas: How wearing the insoles and footwear fits with daily life and thoughts and feelings about wearing insoles and footwear.

1. It would be helpful for me if you could describe what footwear you put on your feet and when during one of your more typical days? Maybe think first about getting out of bed. When do you usually first put on your insoles and shoes for the day. What do you usually wear on your feet during the evening?
2. I’d like to know more about the reasoning behind your daily footwear routine. Please tell me your thoughts? What were you thinking when you got out of bed…when you first put on your insoles and shoes…. when you took off your insoles and shoes. How did you feel?
3. I wonder if you can you think of a place or time when you wouldn’t wear your insoles and shoes? Why is that? Can you tell me more about what you are thinking?
4. What do you feel your family and friends think about your footwear? Does that affect you? Does it change when and where you wear your insoles and shoes?
5. What difference would you say wearing the insoles and footwear has made? To your feet? How you see yourself? How you feel? Why do you think you were given insoles and footwear?
6. Is there anything else you can think of that might influence your decision whether or not to wear your insoles and footwear?
